# Chordin-like 1, a Novel Adipokine, Markedly Promotes Adipogenesis and Lipid Accumulation

**DOI:** 10.3390/cells12040624

**Published:** 2023-02-15

**Authors:** Jinsoo Ahn, Yeunsu Suh, Kichoon Lee

**Affiliations:** Department of Animal Sciences, The Ohio State University, Columbus, OH 43210, USA

**Keywords:** white adipose tissue, adipokine, adipose-specific gene, chordin-like 1 (*Chrdl1*)

## Abstract

White adipose tissue serves as a metabolically dynamic organ that can synthesize and secrete biologically active compounds such as adipokines as well as a caloric reservoir for maintaining energy homeostasis. Adipokines are involved in diverse biological and physiological processes and there have been extensive attempts to characterize the effects of over two dozen adipokines. However, many of these adipokines are produced by not only adipose tissue, but also other tissues. Therefore, investigations into the effects of adipokines on physiological functions have been challenged. In this regard, we aimed to identify a new secreted protein that is encoded by genes specifically expressed in white adipose tissue through analysis of multi-tissue transcriptome and protein expression. As a result, we report a novel adipokine that is encoded by the adipose-specific gene, chordin-like 1 (*Chrdl1*), which is specifically expressed in white adipose tissue in mice; this expression pattern was conserved in the human orthologous *CHRDL1* gene. The expression of *Chrdl1* was enriched in fat cells and developmentally regulated in vitro and in vivo, and moreover, its retrovirus-mediated overexpression and recombinant protein treatment led to markedly increased adipogenesis. Further pathway enrichment analysis revealed enriched pathways related to lipogenesis and adipogenic signaling. Our findings support a pro-adipogenic role of CHRDL1 as a new adipokine and pave the way toward animal studies and future research on its clinical implications and development of anti-obesity therapy.

## 1. Introduction

The global epidemic of obesity is associated with an increased risk of metabolic disorders such as insulin resistance, type II diabetes, hypertension, dyslipidemia, cardiovascular diseases, and cancers [1]. While abnormal accumulation of excessive energy in white adipose tissue (WAT) occurs with obesity, WAT serves, under a normal nutritional state, as a metabolically dynamic endocrine organ that can synthesize and secrete biologically active compounds, including adipokines, as well as a caloric reservoir for maintaining energy homeostasis [2,3]. Expansion of adipose tissue in obesity leads to adipocyte hypertrophy and an increased secretion of pro-inflammatory adipokines, followed by chemokine-driven macrophage accumulation that eventually results in adipose inflammation and insulin resistance [4,5]. Adipose tissue-derived peptide hormones, collectively known as adipokines, are involved in diverse biological and physiological processes and whole-body energy metabolism [6].

There have been extensive attempts to characterize the effects of over two dozen adipokines including the best-known pro-inflammatory leptin and anti-inflammatory adiponectin [7,8,9,10]. However, many of these adipokines are produced by not only adipose tissue, but also other tissues; for example, chemerin has been reported as a hepatokine that is highly produced from the liver as well as an adipokine associated with obesity [11,12]. Therefore, investigations of the effects of adipokines on physiological functions have been challenged. In this regard, further research on secretory proteins that exhibit adipose-specificity and exert its effects on adipogenesis will advance our understanding of the endocrine function of WAT, offering new therapeutic targets for obesity and obesity-related diseases.

In the current study, we report a novel adipokine that is encoded by the adipose-specific gene, chordin-like 1 (*Chrdl1*). The *Chrdl1* gene was specifically expressed in mouse WAT. This WAT-specific expression was conserved in the human orthologous *CHRDL1* gene. Further, the expression of *Chrdl1* was enriched in fat cells and developmentally regulated in vitro and in vivo. Moreover, retrovirus-mediated overexpression of CHRDL in a preadipocyte cell line led to markedly increased adipogenesis, which was also observed dose-dependently with recombinant CHRDL1 protein. Together with transcriptome-based pathway enrichment results showing enriched pathways related to lipogenesis and adipogenic signaling, our findings support a pro-adipogenic role of CHRDL1 as a new adipokine.

## 2. Materials and Methods

### 2.1. Ethics Statement

All animal care and procedures were approved by the Institutional Animal Care and Use Committees (IACUCs) of The Ohio State University (protocol number: 2007A0183).

### 2.2. Sample Preparation

Total RNA was isolated from tissues and cells using Trizol reagent (Invitrogen, Carlsbad, CA, USA) as described in our previous reports [13,14]. Stromal vascular (SV) and fat cell (FC) fractions from iWAT harvested from 1-month-old male Friend leukemia virus-B (FVB) mice (*n* = 3) were separated as described in our previous reports [13]. In brief, the excised tissues were digested in DMEM containing 3.2 mg/mL collagenase II (Sigma-Aldrich) at 37 °C with shaking for 1 h. The digested tissues were then passed through a nylon mesh (100-µm pore size) to eliminate undigested tissue masses and the floating fat cells were separated from the pellet of SV cells by being centrifuged at 500× *g* for 5 min, then SV and FC fractions were collected for RNA extraction. For RNA isolation for in vivo developmental time-points, 2-, 5-, 10-, 24-, and 49-day-old male FVB mice (*n* = 3) were subjected to dissection of iWAT. Mouse proteins were extracted from BAT, iWAT, and epididymal white adipose tissue (eWAT) of 3-month-old male FVB mice (*n* = 3) using lysis buffer as described in our previous report [15]. Human RNAs from the kidney, liver, lung, heart, and muscle tissues were purchased from Agilent Technologies (Santa Clara, CA, USA) and human RNA from WAT was bought from Clontech Laboratories (Mountain View, CA, USA). Human protein extracts from the kidney, liver, lung, heart, muscle, and WAT were obtained from Protein Biotechnologies (Ramona, CA, USA). Samples were stored at −80 °C until use.

### 2.3. Reverse Transcription and Real-Time PCR

These procedures were performed as described in our previous reports [13,16]. In detail, to measure the quantity of RNA, a Nanodrop spectrophotometer (Thermo Scientific, Wilmington, DE, USA) was used, and approximately 1 µg of RNA was reverse-transcribed in a 20 µL total reaction to cDNA using Moloney murine leukemia virus (M-MLV) reverse transcriptase (Invitrogen). The thermal cycle of the reverse transcription was 65 °C for 5 min, 37 °C for 52 min, and 70 °C for 15 min. The quantitative real-time PCR (qPCR) was performed on an ABI 7300 Real-Time PCR instrument (Applied Biosystems, Waltham, MA, USA) by using AmpliTaq Gold polymerase (Applied Biosystems) with SYBR green detection dye. Forward and reverse primers for *Chrdl1* (mF and mR) are listed in Table S1. Reactions were performed in duplicate 25 μL volumes and conditions for the qPCR were 95 °C for 10 min followed by 40 cycles of 94 °C for 15 s, 60 °C for 40 s, 72 °C for 30 s, and 82 °C for 33 s. Relative quantification of gene expression was determined by using the 2^−ΔΔCT^ method [17]. Cyclophilin (*Cyc*) was used as a housekeeping gene (Table S1).

### 2.4. Western Blot Analysis

Western blot analysis with protein extracts was performed as described in our previous report [16]. Briefly, equal amounts of protein lysates were loaded onto gels before wet-transfer to PVDF membranes (Bio-Rad, Hercules, CA, USA). The membranes were blocked for 30 min and then incubated with an appropriate primary antibody at 4 °C overnight. A CHRDL1 polyclonal antibody was raised against an immunogen (EQVKHSDTYCVFQD) in the rabbit by a custom antibody service (Biomatik; Wilmington, DE, USA) after multiple sequence alignments (Figures S1 and S2). The membranes were probed with antibodies against α-tubulin (12G10, DSHB, lowa City, IA, USA), GAPDH (ab9483, Abcam, Cambridge, MA, USA), HA tag (#2367, Cell Signaling Technology, Danvers, MA, USA), FABP4 (AF1443; R & D systems, Minneapolis, MN, USA), C/EBPα (ARP57937; Aviva Systems Biology, San Diego, CA, USA), and PPARγ (LS-C169542; LSBio, WA, USA). The next day, secondary antibodies were applied to the membrane before developing with ECL plus reagents and X-ray films (both materials from GE Healthcare Biosciences, Pittsburgh, PA, USA).

### 2.5. Cell Culture and Adipogenic Differentiation

The 3T3-L1 preadipocytes were differentiated to adipocytes as previously described (Li et al., 2009). In detail, 3T3-L1 preadipocytes (American Type Culture Collection, Manassas, VA, USA) were cultured in DMEM culture media (Invitrogen, Carlsbad, CA, USA) containing 10% fetal bovine serum (Invitrogen) and the mixture solution of penicillin and streptomycin (Pen Strep, 100 units/mL; Invitrogen). The preadipocytes were maintained in the culture media and grown to confluence at 37 °C in 5% CO_2_. At 2 days post-confluence (Day 0), differentiation of growth-arrested 3T3-L1 preadipocytes to adipocytes was induced by treating the preadipocytes with differentiation media, which were the culture media supplemented with 0.25 mM isobutylmethylxanthine (IBMX), 1µM dexamethasone, and 1 µg/mL insulin (Sigma-Aldrich Co., St. Louis, MO, USA). Two days after the induction (Day 2), the differentiation media were changed to the insulin media, which contained 1 µg/mL insulin in the culture media and were maintained for the next 2 days. Two days later (Day 4), the insulin media were changed to the DMEM culture media for another 4 days and the media were changed every 2 days. Total RNA was isolated from the 3T3L1 adipocytes at 0-, 2-, 4-, 6-, and 8-day post-differentiation in vitro. In addition, for the recombinant CHRDL1 treatment, mouse CHRDL1 recombinant protein (LS-G25628; LSBio) was purchased and treated for the indicated time with the indicated concentrations (0–400 ng/mL).

### 2.6. Construction of Retroviral Vectors, Viral Transduction, and Differentiation of Stable Cells

The alternatively spliced short-form of the mouse *Chrdl1* gene was cloned based on the sequence deposited in the NCBI Gene database (GenBank accession no. NM_031258.3) and tagged with hemagglutinin (HA) in the C-terminal region. Forward and reverse primers (mF and mSR-HA) are listed in Table S1. The cloned isoform with the HA tag was subcloned into the retroviral vector pQCXIP (Clontech) which contains the puromycin resistance gene (Pur^r^). The retroviral packaging cell line Ecopack2^TM^-293 (Clontech) was transfected with the pQCXIP expression vector with Lipofectamine 2000 (Invitrogen). After 48 h of transfection, the conditioned media containing retroviruses were collected, filtered, snap-frozen, and stored at −80 °C until use. At the time of retroviral transduction, the conditioned media containing virus particles were supplemented to 3T3-L1 preadipocyte culture media. Then, puromycin-resistant stable 3T3-L1 preadipocytes were selected and induced for differentiation. In particular, the stable cells were plated onto 35-mm petri dishes and incubated until they reached 100% confluent. At 2 days post-confluence (Day 0), the cells were treated with a low-concentration adipogenic cocktail [IBMX (62.5 µM), dexamethasone (0.25 µM), and insulin (1 µg/mL), along with the 10% FBS containing DMEM media] to induce slow and minimal adipogenic differentiation. Two days later (Day 2), the cells were treated with the insulin (1 µg/mL) media for 2 more days and then the media were changed at 2 days interval until day 12.

### 2.7. Staining and Imaging

Oil red O (ORO) staining was performed as described in our previous report [18]. In brief, differentiated 3T3-L1 adipocytes were fixed with 10% formalin for 30 m and replenished with 60% isopropanol. Samples were stained with working ORO solution (0.3%) for 10 min at room temperature. After washing with distilled water, the specimens were dried, and photos were taken. Microscopic images of the samples were captured by an imaging system, AxioCam MRc5 (Carl Zeiss, Oberkochen, Germany). The ORO-stained cells were de-stained with isopropanol and the optical density (OD) of the de-staining isopropanol was measured using a spectrophotometer at a 510 nm wavelength.

### 2.8. Transcriptomic Analysis

For recombinant CHRDL1 treatment, total RNAs were isolated from 3T3-L1 cells at day 3 and sent out for sequencing. RNA-seq was performed by the BGISEQ-500 platform, and after read filtering, cleaned reads were mapped to the mouse reference genome (mm10) using HISAT2 [19]. Bioinformatics analysis including Gene Ontology (GO) and KEGG pathway enrichment analysis were performed with processed data.

### 2.9. Data Mining and Processing

Publicly available datasets used in this study were downloaded from the NCBI Gene Expression Omnibus (GEO) database. These datasets include GSE9954 (microarray for various mouse tissues; alternative accession no. GDS3142) [20], SRP020526 (RNA-seq for various mouse tissues) [21], GSE131861 (RNA-seq of mouse BAT, iWAT, and eWAT) [22], and GSE97205 (RNA-seq for human BAT, sWAT, and oWAT). The raw RNA-seq data were processed as described in our previous report [23]. The processed RNA-seq data from various human tissues were derived from the Genotype-Tissue Expression (GTEx) project (GTEx Analysis V7) and further processed as described in our previous report [24].

### 2.10. Statistical Analysis

Gene expression between two means was compared by a two-tailed Student’s t-test. To test the normality of the data with multiple means, the Shapiro-Wilk normality test was conducted. For non-normal data, the non-parametric Kruskal-Wallis test was performed followed by the Dunn’s post-hoc test. For normal data, one-way ANOVA was conducted followed by the Tukey’s post-hoc test. For the tissue distribution of mRNA expression, a mixed ANOVA model followed by a Fisher’s protected least significant differences was employed using SAS software version 9.4 (SAS Institute. Inc., Cary, NC, USA).

## 3. Results

### 3.1. Expressions of Mouse Chrdl1 Transcripts Were Identified as White Adipose-Specific

**Figure 1:**
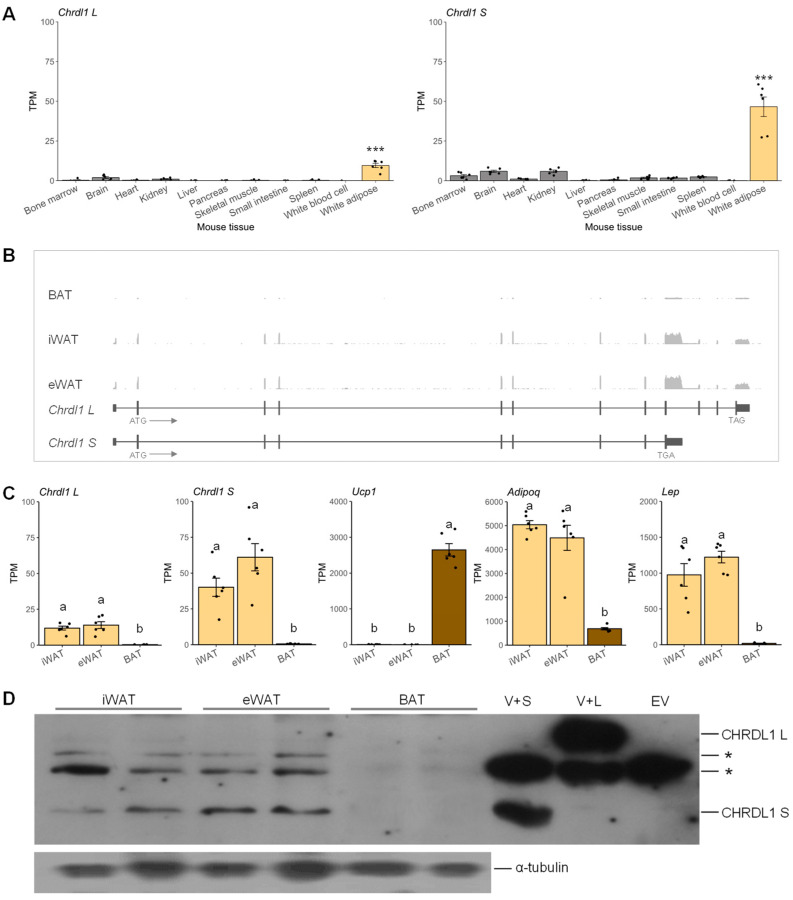
Analysis of *Chrdl1* mRNA and CHRDL1 protein expression levels in mouse tissues. (**A**) The *Chrdl1 L* (long-form) and *Chrdl1 S* (short-form) mRNA expression levels in various mouse tissues. Normalized expression levels in transcripts per million (TPM) values are presented as mean ± SEM. ***, *p* < 0.001. (**B**) Schematic representation of exon and intron structure of the mouse *Chrdl1* gene and the averages of RNA-seq read coverages in BAT, inguinal (iWAT), and epididymal WAT (eWAT) presented in Figure S3B. Arrows denote transcriptional direction. (**C**) The mRNA expression levels of *Chrdl1 L*, *Chrdl1 S*, brown-fat-selective gene (*Ucp1*), and white adipose tissue markers (*Adipoq* and *Lep*) in iWAT, eWAT, and BAT. TPM values are presented as mean ± SEM. Different letters, a and b, denote statistical difference at a significance level of α = 0.05. (**D**) Western blot analysis of CHRDL1 protein expression in mouse white and brown adipose tissues. The experiment was conducted in duplicate. V+S, recombinant vector carrying CHRDL1 short-form protein (CHRDL1 S); V+L, recombinant vector carrying CHRDL1 long-form protein (CHRDL1 L); EV, empty-vector control; *, non-specific band. The α-tubulin protein was used as a loading control.

To identify a novel gene which is predominantly expressed in adipose tissue, we first sorted 45,101 gene expression profiles as described in our previous report [14] and found microarray probes targeting the last exon of *Chrdl1* long-form (1456722_at) and short-form (1434201_at), which were highly ranked as the 277th and 59th adipose-enriched, respectively, out of 45,101 profiles (Table S2). Then, by analyzing RNA-seq data and performing Western blot analysis, the expression of the mouse *Chrdl1* gene at both transcript and protein levels was re-examined. Our analysis of RNA-seq data revealed that the mRNA expression levels of both *Chrdl1* long- and short-forms (*Chrdl1 L* and *Chrdl1 S*, respectively) were significantly higher in white adipose tissue compared to the rest of analyzed tissues (Figure 1A and Figure S3A). The average TPM values of *Chrdl1* long-form (NCBI RefSeq accession no. NM_001358592.1) and short-form (NM_031258.3) were approximately 8-fold and 18-fold higher in WAT (subcutaneous white adipose tissue), respectively, than average TPM values of the rest of tissues (Figure 1A). Next, *Chrdl1* expressions in three different adipose depots, i.e., BAT, inguinal WAT (iWAT), and epididymal WAT (eWAT), were analyzed. The expressions of *Chrdl1 L* and *Chrdl1 S* were almost imperceptible in BAT, based on RNA-seq read coverages in each exon (Figure 1B and Figure S3B). In both iWAT and eWAT, the alternatively spliced *Chrdl1 S* with the premature stop codon (TGA) had an apparently higher expression than *Chrdl1 L*, based on read coverages on the non-overlapping exons (the *Chrdl1 S* last exon *versus* the 10th, 11th, and last exon of *Chrdl1 L*) (Figure 1B and Figure S3B). Upon quantification of the normalized read coverages, approximately 3-fold (in iWAT) and 4-fold (in eWAT) higher expressions of *Chrdl1 S* were shown compared to *Chrdl1 L* (Figure 1C). The mRNA levels of markers displayed their depot-enriched trends: brown-fat-selective gene (*Ucp1*), pan-adipocyte marker (*Adipoq*), white adipose tissue marker (*Lep*) [25], supporting proper sample preparation (Figure 1C). Western blot analysis revealed that the CHRDL1 short-form translation (~37 kDa) occurred prominently in iWAT and eWAT, whereas the CHRDL1 long-form translation was almost absent (Figure 1D). Taken together, *Chrdl1* is a novel WAT-specific gene, and the short transcript (*Chrdl1 S*) was expressed three to four times more than *Chrdl1 L* in WAT and was predominantly translated into the CHRDL1 protein.

### 3.2. Expression of the Human CHRDL1 Gene Was Enriched in White Adipose Tissue

**Figure 2:**
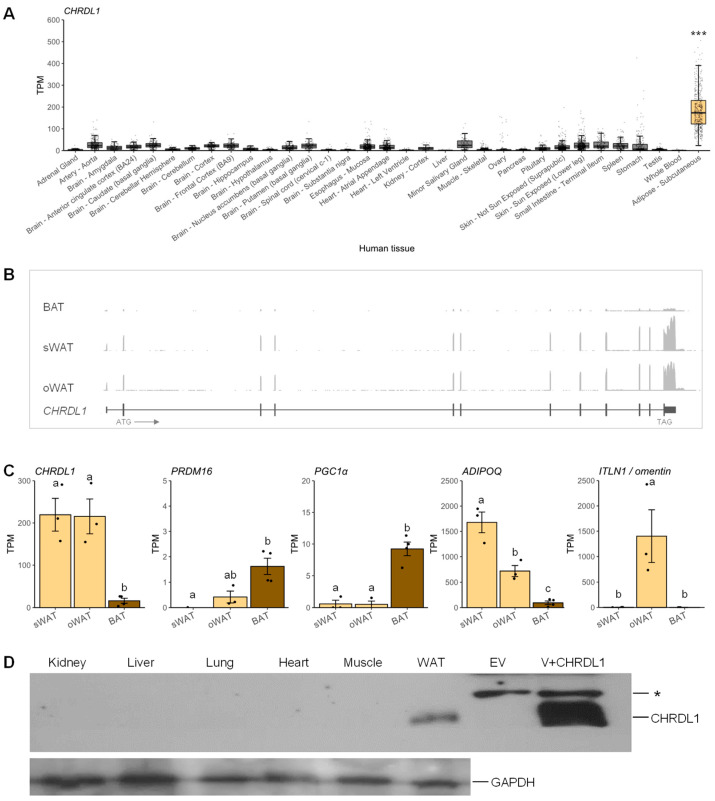
Analysis of *CHRDL1* mRNA and CHRDL1 protein expression patterns in human tissues. (**A**) The *CHDL1* mRNA expression levels in a variety of human tissues. TPM values are presented as mean ± SEM. ***, *p* < 0.001. Each data point is displayed in transparent grey dots due to large sample sizes of the GTEx data (Table S3). The interquartile range shows the middle 50% of values. Whiskers indicate variability outside the upper and lower quartiles. Outliers are shown as data points located outside the whiskers. (**B**) Exon–intron structure of *CHRDL1* with the averages of RNA-seq read coverages in BAT, subcutaneous WAT (sWAT), and omental WAT (oWAT) presented in Figure S4. Transcriptional direction is denoted with an arrow. (**C**) The mRNA expression levels of *CHRDL1*, brown-fat-selective genes (*PRDM16* and *PGC1α*), and white adipose tissue markers (*ADIPOQ* and *CFD/ADIPSIN*) in sWAT, oWAT, and BAT. Mean TPMs ± SEM are presented. Different letters, a, b, and c, represent statistical difference at a significance level of α = 0.05. (**D**) Western blot which is representative of two experiments on CHRDL1 protein expression in human tissues. EV, empty-vector control; V+CHRDL1, recombinant vector carrying human CHRDL1 protein; *, non-specific band. The GAPDH protein was used a loading control.

For comparative analysis, the expression of the human *CHRDL1* gene was further analyzed. By analyzing the GTEx data from human tissue transcriptome studies, a significantly higher expression of *CHRDL1* in subcutaneous white adipose tissue was observed than in the rest of tissues (Figure 2A). In three different adipose depots, i.e., BAT, subcutaneous WAT (sWAT), and omental WAT (oWAT), *CHRDL1* expression was further examined. The expression of the human *CHRDL1* transcript (NM_001143981.2) was very low in BAT; however, it was substantially higher in sWAT and oWAT and there were no detectable alternatively spliced transcripts based on RNA-seq read coverages (Figure 2B and Figure S4). The normalized read coverages were quantified and *CHRDL1* expression was approximately 13-fold higher in both sWAT and oWAT than in BAT (Figure 2C). The brown-fat-selective markers (*PRDM16* and *PGC1α*) were enriched in BAT (Figure 2C) as well as *UCP1* as reported previously [26]. To the contrary, the *ITLN1*/omentin expression was markedly high in oWAT as expected and *ADIPOQ* was enriched in sWAT with lesser degrees in oWAT and BAT (Figure 2C). In addition, the WAT-enriched expression pattern of white adipose tissue marker, *LEP*, was shown in a previous report [26]. Moreover, Western blot analysis disclosed the CHRDL1 protein expression (~52 kDa) in WAT and its deficiency in other human tissues (Figure 2D). In summary, despite the absence of an alternatively spliced isoform in the human, the WAT-specific expression is conserved between the mouse *Chrdl1* gene and the human *CHRDL1* gene at both the transcript and protein levels.

### 3.3. The Chrdl1 Expression Is Enriched in Fat Cells and Regulated during Development In Vitro and In Vivo

**Figure 3:**
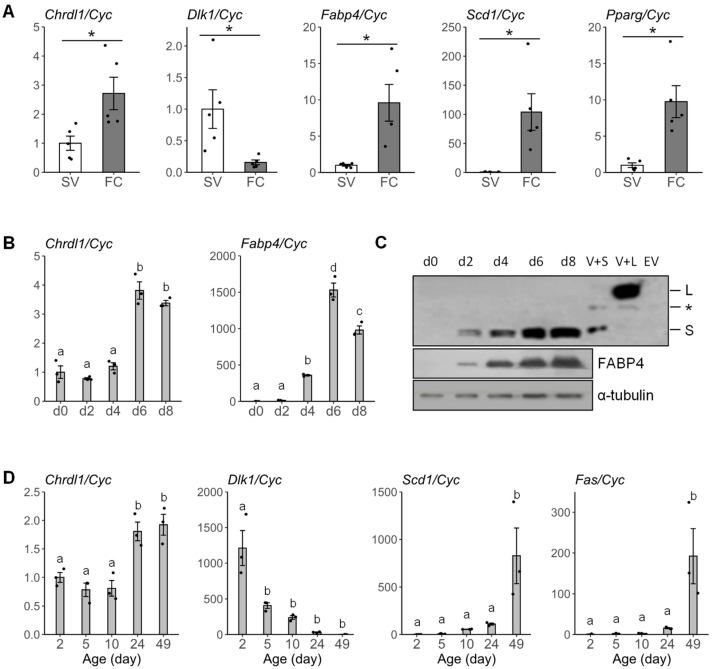
Fat cell-enriched expression of *Chrdl1*, and developmental regulation of *Chrdl1* expression in 3T3-L1 cells and mouse white adipose tissue. (**A**) Relative *Chrdl1* mRNA expression in stromal vascular (SV) and fat cell (FC) fractions from mouse adipose tissue quantified by real-time PCR. *Dlk1* was used a marker for SV enrichment, whereas *Fabp4*, *Scd1*, and *Pparγ* were markers for FC enrichment. (**B**) Relative *Chrdl1* mRNA expression during adipogenic differentiation of 3T3-L1 preadipocytes [day (d) 0 to 8] quantified by real-time PCR. *Fabp4* was used as a marker of adipogenesis. (**C**) Representative western blot of CHRDL1 protein expression during adipogenic differentiation of 3T3-L1 preadipocytes in vitro. The experiment was performed in duplicate. The FABP4 protein was used as a marker for adipogenic differentiation. Coomassie staining and α-tubulin were used as loading controls. V+S, recombinant vector carrying CHRDL1 short-form protein (CHRDL1 S); V+L, recombinant vector carrying CHRDL1 long-form protein (CHRDL1 L); EV, empty-vector control; *, non-specific band. (**D**) Relative *Chrdl1* mRNA expression during development of iWAT in vivo (in 2-day to 49-day-old mice) quantified by real-time PCR. *Dlk1* was used as a negative marker of adipose development, while *Scd1* and *Fas* were positive markers of adipose development. The *Cyc* gene was used as a reference gene. Values represent quadruplicate means ± SEM. *, *p* < 0.05. Different letters, a, b, c and d, indicate statistical difference at a significance level of α = 0.05.

To investigate expression levels of *Chrdl1* in adipocytes, stromal vascular (SV) and fat cell (FC) fractions were derived from the iWAT of the mouse, and the fractionation was verified by predominant expression of *Dlk1* in SV cells, and *Fabp4*, *Scd1*, and *Pparγ* in fat cells (*p* < 0.05) (Figure 3A). In SV cells, *Chrdl1* mRNA was expressed at low levels. In FCs, the expression level of *Chrdl1* mRNA was approximately 2.8-fold higher compared to the level in SV cells (*p* < 0.05) (Figure 3A). These expression patterns indicate that the mouse *Chrdl1* gene is highly expressed in fat cells (adipocytes). During adipogenic differentiation of the 3T3-L1 preadipocyte cell line in vitro, the *Chrdl1* mRNA expression was significantly increased at day 6 post-differentiation and maintained until day 8 (Figure 3B). Western blot analysis revealed that the mouse CHRDL1 S protein expression was also increased during the differentiation and strong expression signals were detected at day 6 and 8, while the CHRDL1 L protein was unnoticeable (Figure 3C). To further examine whether the progressive expression pattern of *Chrdl1* is also observed in vivo, expression was analyzed with samples from iWAT collected during the development of mice. Expressions of the adipogenic markers, *Scd1* and *Fas*, were significantly increased at day 49 during the pubertal period, and expression of the preadipocyte marker, *Dlk1*, was significantly decreased at day 5 during the neonatal stage (Figure 3D). The expression level of *Chrdl1* was significantly increased approximately two-fold at day 24 (after weaning) and maintained until day 49 (Figure 3D). Collectively, in addition to enrichment in fat cells, the expression of *Chrdl1* mRNA and CHRDL1 protein increased during adipocyte development in vitro and/or in vivo, suggesting the potential role of the *Chrdl1* gene in adipogenesis.

### 3.4. Overexpression of Chrdl1 Increased Adipogenic Differentiation of 3T3-L1 Preadipocytes

**Figure 4:**
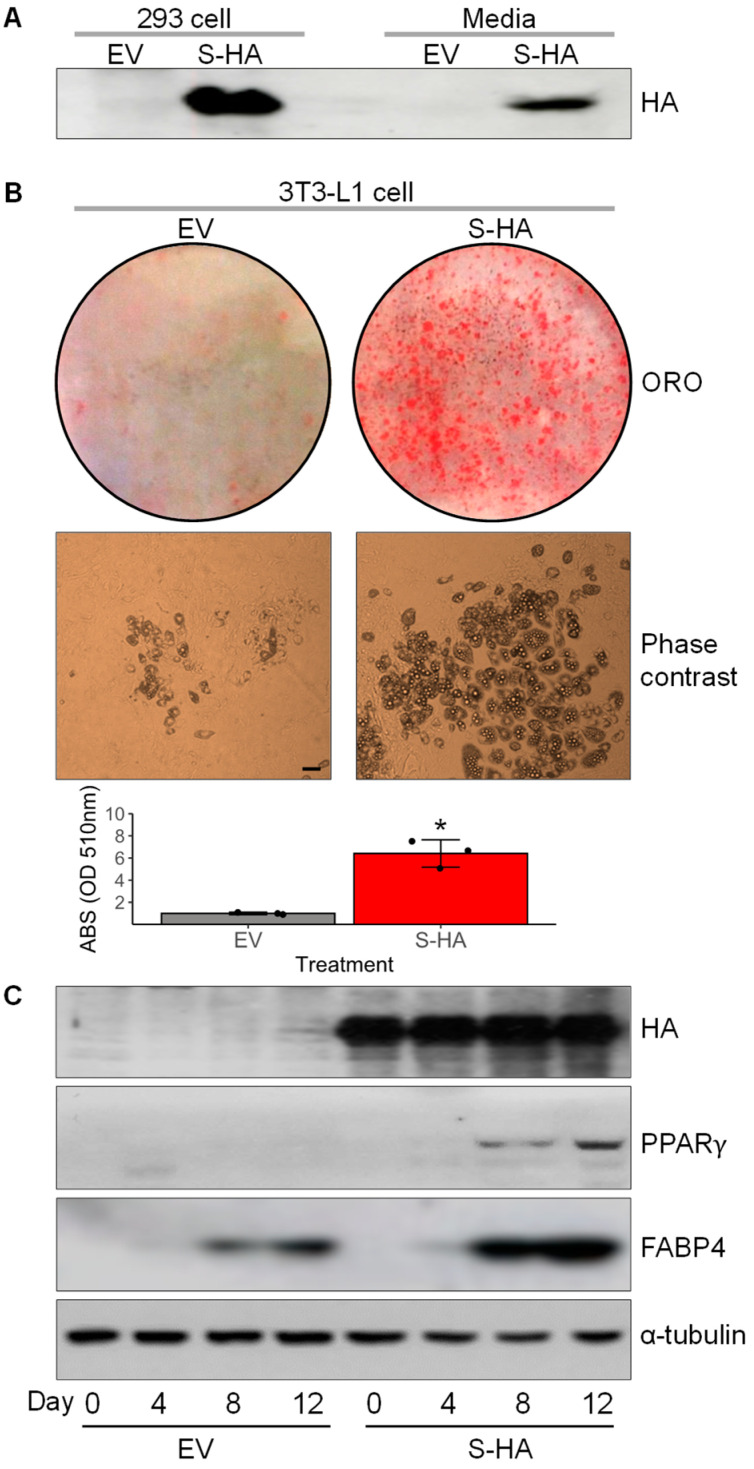
Effects of overexpression of *Chrdl1* in adipogenic differentiation of 3T3-L1 preadipocytes. (**A**) Representative Western blots of HA-tagged CHRDL1 short-form (S-HA) in 293 cell lysates and culture media after transfection. The experiment was performed in triplicate. (**B**) Oil Red O staining and phase-contrast images of S-HA overexpressing 3T3-L1 cells. For quantification, absorbance (ABS) was measured for the ORO-stained lipid droplets at 510 nm. Values are the mean ± SEM of three independent experiments. *, *p* < 0.05. (**C**) Representative Western blots of overexpressed S-HA protein detected against an anti-HA tag antibody and positive markers of adipogenic differentiation and lipid accumulation (PPARγ and FABP4) in 3T3-L1 cells. The experiment was conducted in duplicate. Cell lysates were collected at day 0, 4, 8, and 12 after induction of adipogenic differentiation. The α-tubulin protein was used as a loading control. EV, empty vector that was transfected as a control. The scale bar indicates 100 µm.

To investigate the effect of *Chrdl1* on adipocyte differentiation, 3T3-L1 preadipocytes were transduced with recombinant retroviruses producing CHRDL1. For this purpose, retroviruses having a vector fused with HA-tagged *Chrdl1 S* (S-HA) or an empty vector (EV) were generated using Ecopack2^TM^-293 cells followed by Western blotting with an anti-HA tag antibody for confirmation, and the secretion of S-HA was further detected in cell culture media as expected by signal peptide prediction with SignalP-5.0 [27] (Figure 4A and Figure S1). After the transduction of 3T3-L1 preadipocytes with the retroviruses and selection of puromycin-resistant stable cells, differentiation was induced minimally with the low-concentration adipogenic cocktail to avoid effects of adipogenic inducers that can override CHRDL1 effect and to obtain maximum contrast in the degrees of differentiation between the two treatments. At day 12 post-differentiation, S-HA overexpressing cells displayed markedly greater lipid accumulation (approximately 6-fold higher) and a significantly increased number of adipocytes in Oil Red O staining and phase contrast microscopy, respectively, compared to EV transfected cells (Figure 4B). The adipogenic differentiation was confirmed by PPARγ and FABP4 protein expression at Days 8 and 12 post-differentiation (Figure 4C). The S-HA overexpressing cells exhibited higher PPARγ and FABP4 protein expression levels than EV transfected cells, indicating greater adipogenic differentiation. Of note, the S-HA protein expression level was relatively consistent during differentiation, and it was due possibly to the effect of a strong CMV promoter in the retroviral vector (Figure 4C). Overall, the mouse CHRDL1 short-form overexpression resulted in an increased adipocyte differentiation and lipid accumulation, implying the role of CHRDL1 as a candidate pro-adipogenic adipokine.

### 3.5. Recombinant CHRDL1 Protein Increased Both Induced and Spontaneous Adipogenic Differentiation Dose-Dependently

**Figure 5:**
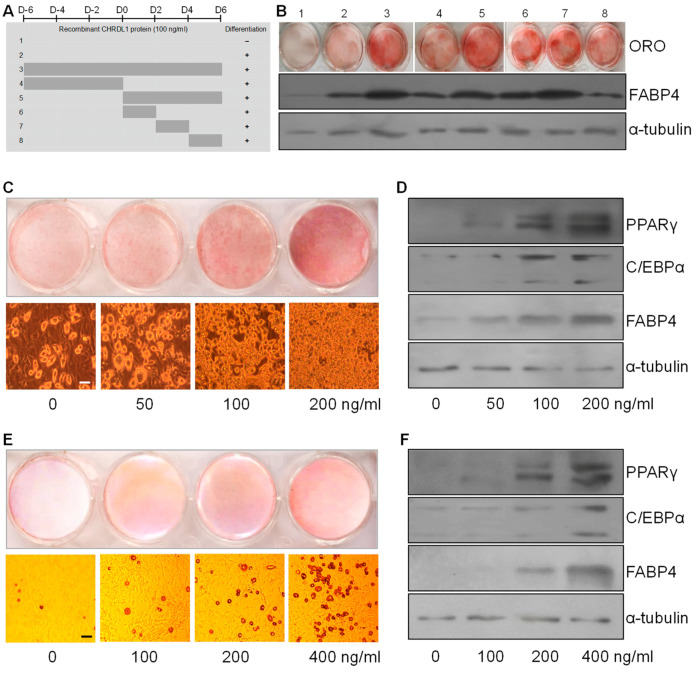
Induced and spontaneous adipogenic differentiation of 3T3-L1 cells with administration of CHRDL1 recombinant protein. (**A**) Schematic timetable of the search for a permissive stage of differentiation by CHRDL1. Adipogenic differentiation without (treatment 1 and 2) or with different periods of CHRDL1 recombinant protein treatment (100 ng/mL) denoted by grey parallel bars (treatment 3 through 8). D0, time of differentiation induction which was preceded or followed by 2-day intervals. −, no induction of differentiation at D0; +, induction of differentiation at D0. (**B**) Oil Red O staining and Western blots of the adipogenic marker FABP4 at day 8 (D8) for each treatment in A. (**C**) Dose-dependent effects of administered CHRDL1 recombinant protein on induced 3T3-L1 adipocyte differentiation and lipid accumulation. The experiment was performed in duplicate. (**D**) Western blots of adipogenic markers with the proteins harvested from C. (**E**) Spontaneous differentiation of 3T3-L1 preadipocytes along with administration of indicated doses of CHRDL1 recombinant protein. The experiment was performed in duplicate. (**F**) Western blot analysis of adipogenic markers for the proteins collected from E. The α-tubulin protein was used as a loading control. Scale bars indicate 100 µm.

For the purpose of determining a permissive stage for adipogenic differentiation by CHRDL1 and its dose-dependent effects, recombinant CHRDL1 protein was administered to the 3T3-L1 cell media. By comparing different periods for CHRDL1 recombinant protein treatment (100 ng/mL) which were either throughout, prior to, or after the time of differentiation induction (D0) (Figure 5A), the permissive stage for adipogenic differentiation by CHRDL1 was ascertained. Among the treatments 1 through 3, treatment 3 with both differentiation induction and CHRDL1 recombinant protein showed much more lipid accumulation and expression of adipocyte differentiation marker protein (FABP4) at day 8 (D8) (Figure 5B). Then, the period of treatment 3 was divided into periods for the treatment 4 and 5, and, between treatment 4 and 5, substantially more lipid accumulation and expression of FABP4 were observed with treatment 5, suggesting a stimulatory effect of CHRDL1 recombinant protein on adipocyte differentiation after the induction of differentiation at D0 (Figure 5B). To narrow it down, the period of treatment 5 was divided into periods for treatments 6 through 8, and, among treatments 6–8, treatment 6 and 7 showed considerably higher levels of lipid accumulation and FABP4 expression, implying the period of D0 through D4 is the permissive stage of 3T3-L1 differentiation by recombinant CHRDL1 protein (Figure 5B). Therefore, at 4 days of differentiation, the treatment during the permissive stage (D0–D4) with four different concentrations of recombinant CHRDL1 protein were evaluated, and it was shown that the lipid accumulation increased very strongly depending on the dose (Figure 5C). Further, the expression adipocyte differentiation markers (PPARγ, C/EBPα, and FABP4) increased markedly and dose-dependently (Figure 5D). Together, it suggested that CHRDL1 mediated such a strong and fast promotion of adipogenesis, which has never been reported to the best of our knowledge. On a next evaluation, spontaneous differentiation with higher concentrations also led to an increased adipogenic differentiation in a dose-dependent manner, consolidating the potential pro-adipogenic role of CHRDL1 (Figure 5E,F). In conclusion, the effects of CHRDL1 on adipocyte differentiation were assessed temporally and dose-dependently, providing evidence of a strong stimulatory effect of CHRDL1 on adipogenesis.

### 3.6. Transcriptomic Analysis Revealed That CHRDL1 Up-Regulates Signaling Pathways Related to Adipogenesis

**Figure 6:**
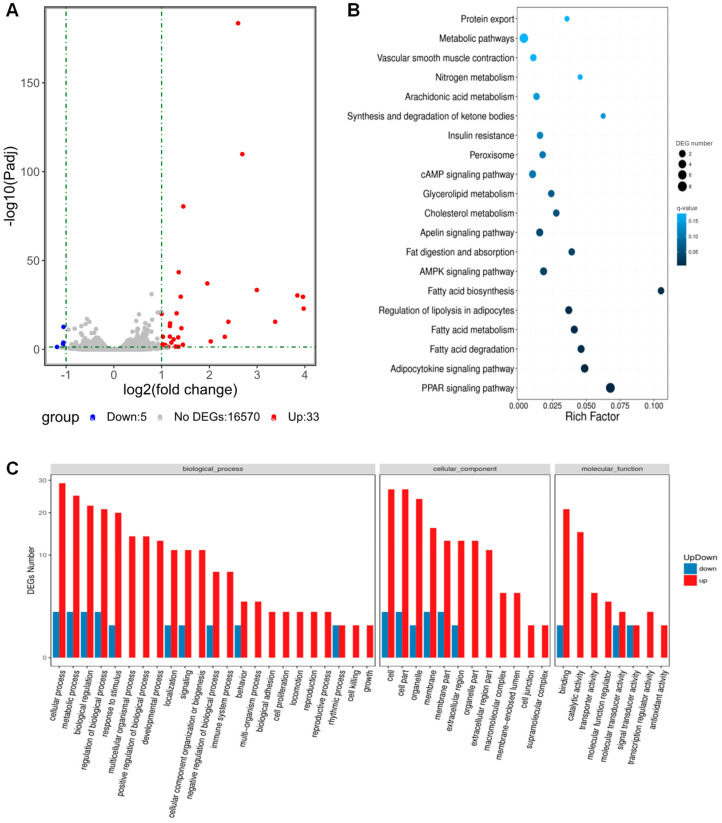
RNA-seq of 3T3-L1 preadipocytes treated with recombinant CHRDL1 protein. (**A**) Volcano plot of differentially expressed genes (DEGs) between treated samples (with recombinant CHRDL1 protein, 100 ng/mL) and untreated controls. Sequencing was performed in triplicates. The *x*-axis represents log2 transformed fold change. The *y*-axis represents −log10 transformed adjusted *p*-value (*P*adj), and the larger values indicate more significance. DEGs passed a threshold of |fold change| > 2 and *P*adj < 0.05 (green dash-dotted lines). Up, up-regulated DEGs in treated samples; Down, down-regulated DEGs in treated samples; NoDEGs, genes that are not differentially expressed. (**B**) Enriched KEGG pathways. The color indicates the *q*-value, and the lower *q*-value (darker) indicates the more significant enrichment. The point size indicates DEG numbers. Rich Factor is a value of enrichment factor which is obtained by dividing the number of DEGs by the total number of genes in the corresponding pathway. The larger the Rich Factor, the more the significant enrichment. (**C**) Gene Ontology (GO) enrichment analysis. The *x*-axis represents the most enriched GO terms in the categories of biological processes, cellular components, and molecular functions. The *y*-axis represents the number of up- or down-regulated DEGs.

To further investigate whether CHRDL1 is related to cell signaling pathways regulating adipogenesis, we performed RNA-seq using samples prepared with or without the recombinant CHRDL1 treatment (100 ng/mL) and on day 3 when the 3T3-L1 cells were differentiating after mitotic clonal expansion (two rounds of mitosis) for 2 days post differentiation induction [28] (Figure S5). Among 16,608 analyzed genes, there were 33 up-regulated differentially expressed genes (DEGs) and 3 down-regulated DEGs, with a threshold of an absolute value of fold change > 2 and adjusted *p*-value < 0.05 (Figure 6A, Table S4). The KEGG pathway analysis showed that a signaling pathway with the highest rich factor (enrichment factor) was ‘Fatty acid biosynthesis’ which consisted of genes encoding acyl-CoA synthetases (*Acsl1* and *Acsbg1*; both were up-regulated.) (Figure 6B). Further, a signaling pathway with the lowest *q*-value (3.48 × 10^−11^) was the ‘PPAR signaling pathway’, which is composed of all up-regulated genes (*Adipoq*, *Fabp4*, *Cd36*, *Cpt1a*, *Acsl1*, *Hmgcs2*, *Fabp5*, *Angptl4*, *Acsbg1*, and *Plin1*) that are involved in adipogenesis (Figure 6B). Generally, the lesser the enrichment factor, the higher the *q*-value (lesser significance). In addition, gene ontology (GO) analysis showed that many up-regulated DEGs are involved in cellular developmental processes (Figure 6C). In summary, the most significantly enriched pathways under CHRDL1 treatment were related to lipogenesis and adipogenic signaling.

## 4. Discussion

Here, we report a newly identified adipokine CHRDL1 that is encoded by the white adipose-enriched *Chrdl1* gene. Through extensive comparison of various mouse and human tissues using transcriptome analysis and Western blot analysis, the WAT-enriched expression pattern of *Chrdl1*, the orthologous human *CHRDL* gene, and their encoded protein CHRDL1 were revealed, suggesting their potential roles in adipocyte biology. Both mouse and human CHRDL1 were detected as a single dominant isoform in WAT. Noticeably, unlike *Adipoq* which is expressed in both white and brown adipocytes [29], expressions of the *Chrdl1* gene and its encoded protein CHRDL1 was almost undetectable in BAT. Further, unlike *ITLN1* (omentin-1) which is specific to omental fat, but not subcutaneous fat, in the human [30], *CHRDL1* expression levels were significantly high in both oWAT and sWAT while undetectable in BAT. Identifying the adipose tissue-enriched genes that are expressed predominantly in adipose tissue and further determining secreted protein-encoding genes among those genes have been conducted during candidate novel adipokine research [7,8,9,30]. In addition, regarding adipocyte differentiation, a slight enhancement has been reported with adipokine vaspin treatment [31]. However, as reported in this study, detection of the strong and fast promotion of adipogenesis will be important for targeting critical adipokines for downstream analysis.

As 3T3-L1 preadipocyte cell lines are considered an appropriate in vitro model for investigating adipocyte secretome [32], the secretion of CHRDL1 to the 3T3-L1 cell culture media was confirmed in this study. The previous estimate of total number of proteins in secretome reached approximately 2200 which is about 7% of a total 30,000 human proteins and many secretory proteins are expressed from multiple tissues and organs [33,34]. Considering the intricacies of multi-organ systems, developing tools for identifying tissue-specific secretory proteins is of particular interest and ongoing [35]. Because *Chrdl1* is specifically expressed in WAT, investigations on the effects of secreted CHRDL1 will advance our understanding on adipokines predominantly derived from WAT. Further, the developmental expression pattern described above showed that the CHRDL1 expression was gradually increased during the differentiating stage of the 3T3-L1 preadipocytes and maintained at a fully differentiated stage (mature adipocytes), implying its role during adipocyte development. Retrovirus-mediated overexpression of CHRDL1 in the 3T3-L1 preadipocyte cell line and recombinant CHRDL protein treatments provided evidence for the previously unrecognized effect of CHRDL on adipogenesis. The CHRDL1 produced in adipocytes may affect adipocytes themselves and/or neighboring cells in autocrine and/or paracrine manners, or CHRDL1 from mature adipocytes may trigger adipogenic differentiation of nearby preadipocytes which might be potential action mechanisms in adipose tissue in vivo.

The pathogenic effects related to obesity are greatly attributed to dysregulated secretion of adipokines, which leads to decreased insulin sensitivity (insulin resistance) in peripheral tissues including skeletal muscle and the liver [4]. Therefore, adipokines are promising candidates for the future treatment of obesity and obesity-related diseases. As indicated in the aforementioned pathway analysis, acyl CoA synthetases were up-regulated with CHRDL1 treatment and could convert free fatty acids to fatty acyl-CoA esters, and thereby play a key role in lipid biosynthesis [36]. Interestingly, reduced concentration of plasma lipids was recently reported to be strongly correlated with increased *CHRDL1* expression [37]. Also, supplementary data from a previous study on sex and aging plasma proteomes showed that mouse plasma CHRDL1 did not change with sex whereas it was significantly up-regulated with age (*q* = 8.57 × 10^−4^) [38] This increased circulating level of CHRDL1 during aging was conserved in humans (*q* = 8.36 × 10^−38^) and consistent with previous results [39]. In this regard, interactions between CHRDL1 levels, age, and plasma lipids that might affect cardiometabolic health should be further investigated. In addition, as experimental perspectives, for the induction of differentiation, a reduced concentration of IBMX and dexamethasone was used to show a more drastic effect of CHRDL on the differentiation, and it led to very low lipid accumulation in control cells and much higher lipid accumulation in CHRDL1 overexpressing cells. In addition, the HA-tag was used to distinguish endogenous CHRDL1 expression and overexpressed CHRDL expression. Further, for future research, expressions of binding partners in white adipose tissue (autocrine/paracrine signaling) or other tissues (endocrine signaling) need to be identified and analyzed. Overall, our findings support the pro-adipogenic role of CHRDL1 as a new adipokine and pave the way toward animal studies and future research on its clinical implications and development of anti-obesity therapy.

## Data Availability

The data generated and processed during this study can be found within the manuscript and Supplementary Materials. Publicly available datasets processed in this study include GSE9954 (microarray for mouse tissues), GSE132040 (RNA-seq for 3-month-old mouse tissues), GSE131861 (RNA-seq for mouse BAT, iWAT, and eWAT), GSE97205 (RNA-seq for human BAT, sWAT, and oWAT), and GTEx Analysis V7 (processed RNA-seq data for human tissues).

## Supplementary Materials

The following supporting information can be downloaded at: https://www.mdpi.com/article/10.3390/cells12040624/s1, Figure S1: Amino acid sequence analysis of CHRDL1; Figure S2: Multiple alignment of chordin-like 1 nucleotide sequences; Figure S3: Expression levels of mouse *Chrdl1* mRNA; Figure S4: Expression levels of human *CHRDL1* mRNA; Figure S5: Measurement of cell numbers; Table S1: Primer sequences for real-time PCR and cloning; Table S2: Microarray-based mouse transcriptome dataset; Table S3: Sample sizes of the GTEx data for human tissues. Table S4: RNA-seq analysis of 3T3-L1 cells with or without CHRDL1 treatment.
