# Supplementary material for: Chordin-like 1, a Novel Adipokine, Markedly Promotes Adipogenesis and Lipid Accumulation
Source: Cells. 2023 Feb 15;12(4):624. doi: 10.3390/cells12040624 (PMC9953935; doi:10.3390/cells12040624)
Supplement: Supplementary file 1 [file cells-12-00624-s001.zip › Supplementary_Figures.pdf]

A

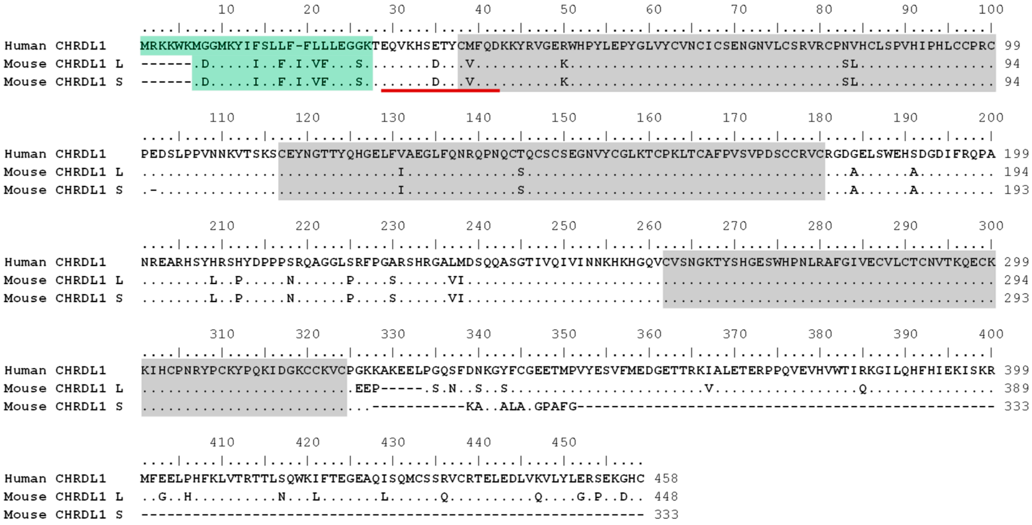
Human CHRDL1
Mouse CHRDL1 L
Mouse CHRDL1 S

B

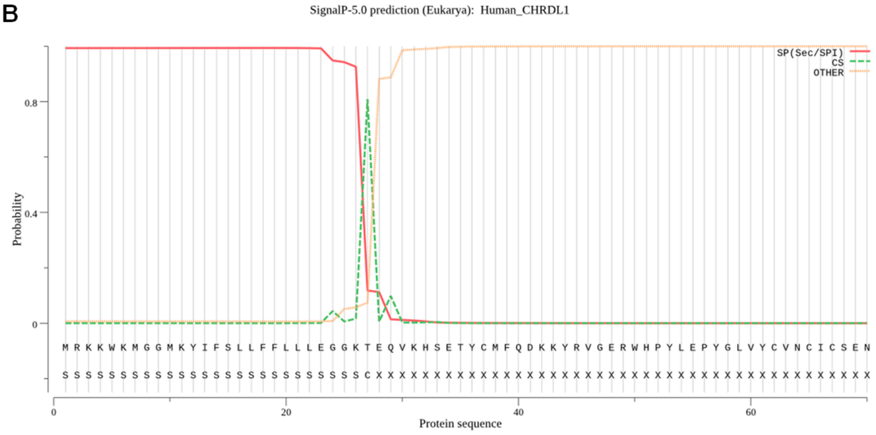
SignalP-5.0 prediction (Eukarya): Human_CHRDL1
SP(Sec/SPI)
CS
OTHER
Probability
Protein sequence

C

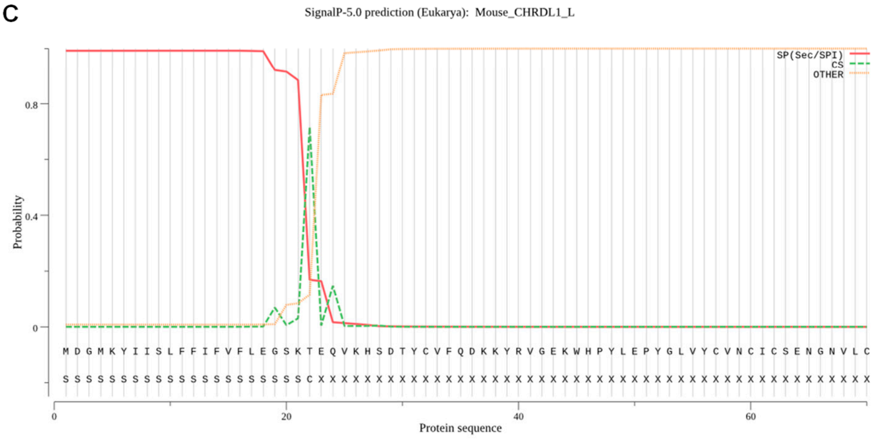
SignalP-5.0 prediction (Eukarya): Mouse_CHRDL1_L
SP(Sec/SPI)
CS
OTHER
Probability
Protein sequence

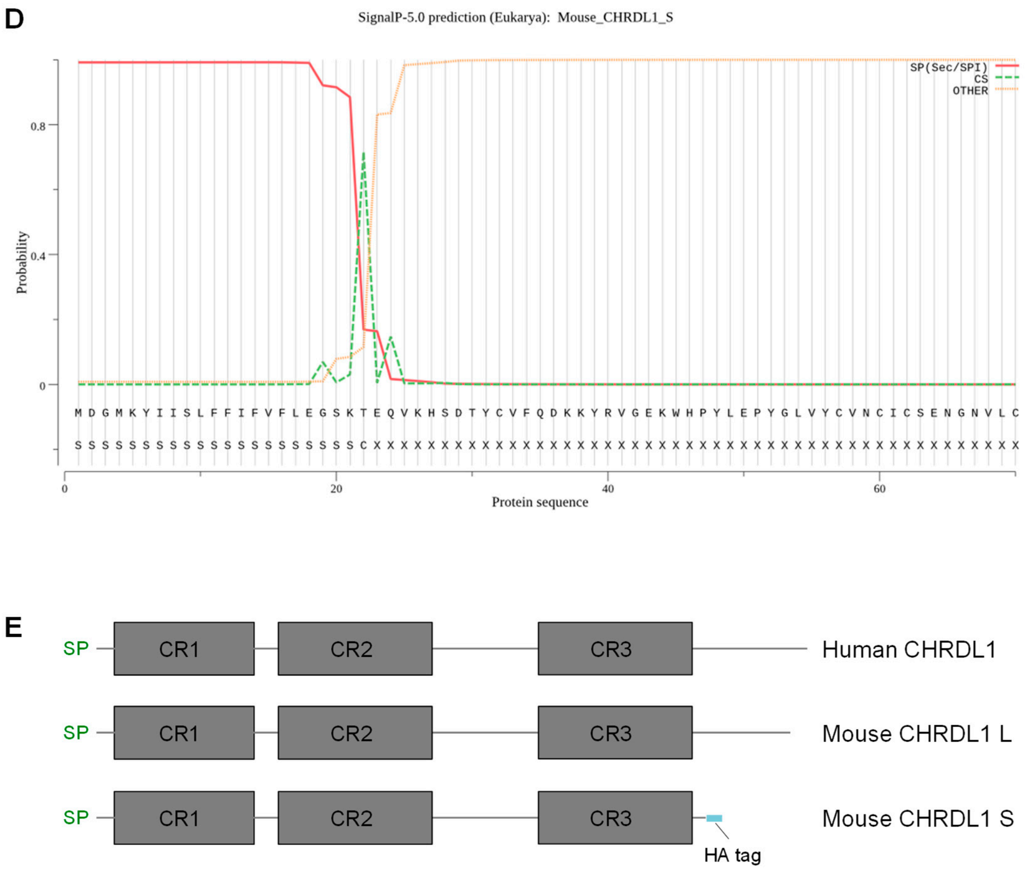

Figure S1. Amino acid sequence analysis of CHRDL1. (A) Multiple alignment of human and mouse CHRDL1 proteins. Predicted signal peptides are highlighted with green. Three CR domains are highlighted with grey. An immunogen (EQVKHSDTYCVFQD) for anti-CHRDL1 antibody is underlined in red. (B-D) SignalP-5.0 was used for prediction of signal peptides for human CHRDL1 (B), mouse CHRDL1 long-form (C), and mouse CHRDL1 short-form (D). (E) Schematic diagram of CHRDL1 proteins. HA-tag for the fused S-HA protein is denoted in mouse CHRDL1 S. SP, signal peptide; CR, cysteine-rich domain.

```
                        10        20        30        40        50        60        70        80        90       100
               ....|....|....|....|....|....|....|....|....|....|....|....|....|....|....|....|....|....|....|....|
Human CHRDL1   ATGAGAAAAAAGTGGAAAATGGGAGGCATGAAATACATCTTTTCGTTGTTGTTC---TTTCTTTTGCTAGAAGGAGGCAAAACAGAGCAAGTAAAACATT 97
Mouse Chrdl1 L ------------------....AT...............A....C..A..T...ATC...G....C.........A..........A...........C. 82
Mouse Chrdl1 S ------------------....AT...............A....C..A..T...ATC...G....C.........A..........A...........C. 82

                       110       120       130       140       150       160       170       180       190       200
               ....|....|....|....|....|....|....|....|....|....|....|....|....|....|....|....|....|....|....|....|
Human CHRDL1   CAGAGACATATTGCATGTTTCAAGACAAGAAGTACAGAGTGGGTGAGAGATGGCATCCTTACCTGGAACCTTATGGGTTGGTTTACTGCGTGAACTGCAT 197
Mouse Chrdl1 L ....C.........G...................T.............A.........C...........G....AC...........T........... 182
Mouse Chrdl1 S ....C.........G...................T.............A.........C...........G....AC...........T........... 182

                       210       220       230       240       250       260       270       280       290       300
               ....|....|....|....|....|....|....|....|....|....|....|....|....|....|....|....|....|....|....|....|
Human CHRDL1   CTGCTCAGAGAATGGGAATGTGCTTTGCAGCCGAGTCAGATGTCCAAATGTTCATTGCCTTTCTCCTGTGCATATTCCTCATCTGTGCTGCCCTCGCTGC 297
Mouse Chrdl1 L ......T........................................G.C.............A..C.................C..T.....C...... 282
Mouse Chrdl1 S ......T........................................G.C.............A..C.................C..T.....C...... 282

                       310       320       330       340       350       360       370       380       390       400
               ....|....|....|....|....|....|....|....|....|....|....|....|....|....|....|....|....|....|....|....|
Human CHRDL1   CCAGAAGACTCCTTACCCCCAGTGAACAATAAGGTGACCAGCAAGTCTTGCGAGTACAATGGGACAACTTACCAACATGGAGAGCTGTTCGTAGCTGAAG 397
Mouse Chrdl1 L ....---..........A.............................A.....A........A..C.................A......A......... 379
Mouse Chrdl1 S ....---..........A.............................A.....A........A..C.................A......A......... 379

                       410       420       430       440       450       460       470       480       490       500
               ....|....|....|....|....|....|....|....|....|....|....|....|....|....|....|....|....|....|....|....|
Human CHRDL1   GGCTCTTTCAGAATCGGCAACCCAATCAATGCACCCAGTGCAGCTGTTCGGAGGGAAACGTGTATTGTGGTCTCAAGACTTGCCCCAAATTAACCTGTGC 497
Mouse Chrdl1 L .............C..............G....GT.....T.....C........G..T..A..C........................C.G........ 479
Mouse Chrdl1 S .............C..............G....GT.....T.....C........G..T..A..C........................C.G........ 479

                       510       520       530       540       550       560       570       580       590       600
               ....|....|....|....|....|....|....|....|....|....|....|....|....|....|....|....|....|....|....|....|
Human CHRDL1   CTTCCCAGTCTCTGTTCCAGATTCCTGCTGCCGGGTATGCAGAGGAGATGGAGAACTGTCATGGGAACATTCTGATGGTGATATCTTCCGGCAACCTGCC 597
Mouse Chrdl1 L A.......................T........A...........G....C....T.A..G.........G.G........................... 579
Mouse Chrdl1 S A.......................T........A...........G....C....T.A..G.........G.G........................... 579

                       610       620       630       640       650       660       670       680       690       700
               ....|....|....|....|....|....|....|....|....|....|....|....|....|....|....|....|....|....|....|....|
Human CHRDL1   AACAGAGAAGCAAGACATTCTTACCACCGCTCTCACTATGATCCTCCACCAAGCCGACAGGCTGGAGGTCTGTCCCGCTTTCCTGGGGCCAGAAGTCACC 697
Mouse Chrdl1 L .........................T...T..C.C...C..............A.A....A..........TC..............AG........... 679
Mouse Chrdl1 S .........................T...T..C.C...C..............A.A....A..........TC..............AG........... 679

                       710       720       730       740       750       760       770       780       790       800
               ....|....|....|....|....|....|....|....|....|....|....|....|....|....|....|....|....|....|....|....|
Human CHRDL1   GGGGAGCTCTTATGGATTCCCAGCAAGCATCAGGAACCATTGTGCAAATTGTCATCAATAACAAACACAAGCATGGACAAGTGTGTGTTTCCAATGGAAA 797
Mouse Chrdl1 L ........G....A.................C..G.....C.....G.................G.....A............................. 779
Mouse Chrdl1 S ........G....A.................C..G.....C.....G.................G.....A............................. 779

                       810       820       830       840       850       860       870       880       890       900
               ....|....|....|....|....|....|....|....|....|....|....|....|....|....|....|....|....|....|....|....|
Human CHRDL1   GACCTATTCTCATGGCGAGTCCTGGCACCCAAACCTCCGGGCATTTGGCATTGTGGAGTGTGTGCTATGTACTTGTAATGTCACCAAGCAAGAGTGTAAG 897
Mouse Chrdl1 L ......C........A.................T..A..A.................A.....A.....C.......................A...... 879
Mouse Chrdl1 S ......C........A.................T..A..A.................A.....A.....C.......................A...... 879

                       910       920       930       940       950       960       970       980       990      1000
               ....|....|....|....|....|....|....|....|....|....|....|....|....|....|....|....|....|....|....|....|
Human CHRDL1   AAAATCCACTGCCCCAATCGATACCCCTGCAAGTATCCTCAAAAAATAGACGGAAAATGCTGCAAGGTGTGTCCAGGTAAAAAAGCAAAAGAAGAACTTC 997
Mouse Chrdl1 L ..................................................T.....G..............C...AAG..CCTC...GCC...A.C---- 975
Mouse Chrdl1 S ..................................................T.....G..............C...........G.......GT.C.---- 975

                      1010      1020      1030      1040      1050      1060      1070      1080      1090      1100
               ....|....|....|....|....|....|....|....|....|....|....|....|....|....|....|....|....|....|....|....|
Human CHRDL1   CAGGCCAAAGCTTTGACAATAAAGGCTACTTCTGCGGGGAAGAAACGATGCCTGTGTATGAGTCTGTATTCATGGAGGATGGGGAGACAACCAGAAAAAT 1097
Mouse Chrdl1 L -----------......GC......T.C...T..T..A........C........A...........G..............A...............G. 1064
Mouse Chrdl1 S -----------..G.CTGGAGGCCCTGC...TG.TT.A-------------------------------------------------------------- 1002

                      1110      1120      1130      1140      1150      1160      1170      1180      1190      1200
               ....|....|....|....|....|....|....|....|....|....|....|....|....|....|....|....|....|....|....|....|
Human CHRDL1   AGCACTGGAGACTGAGAGACCACCTCAGGTAGAGGTCCACGTTTGGACTATTCGAAAGGGCATTCTCCAGCACTTCCATATTGAGAAGATCTCCAAGAGG 1197
Mouse Chrdl1 L ............C..............A.........................A........................C...........T......... 1164
Mouse Chrdl1 S ---------------------------------------------------------------------------------------------------- 1002

                      1210      1220      1230      1240      1250      1260      1270      1280      1290      1300
               ....|....|....|....|....|....|....|....|....|....|....|....|....|....|....|....|....|....|....|....|
Human CHRDL1   ATGTTTGAGGAGCTTCCTCACTTCAAGCTGGTGACCAGAACAACCCTGAGCCAGTGGAAGATCTTCACCGAAGGAGAAGCTCAGATCAGCCAGATGTGTT 1297
Mouse Chrdl1 L .......G......C.A...T........A..T..TC.G..C...T...A..........C.......T...............C.............C. 1264
Mouse Chrdl1 S ---------------------------------------------------------------------------------------------------- 1002

                      1310      1320      1330      1340      1350      1360      1370      1380
               ....|....|....|....|....|....|....|....|....|....|....|....|....|....|....|....|
Human CHRDL1   CAAGTCGTGTATGCAGAACAGAGCTTGAAGATTTAGTCAAGGTTTTGTACCTGGAGAGATCTGAAAAGGGCCACTGTTAG 1377
Mouse Chrdl1 L ......AG..G..............G............C...............G....C.........A.......... 1344
Mouse Chrdl1 S -------------------------------------------------------------------------------- 1002
```

Figure S2. Multiple alignment of chordin-like 1 nucleotide sequences. Human *CHRDL1* and mouse *Chrdl1* protein-coding nucleotide sequences.

A

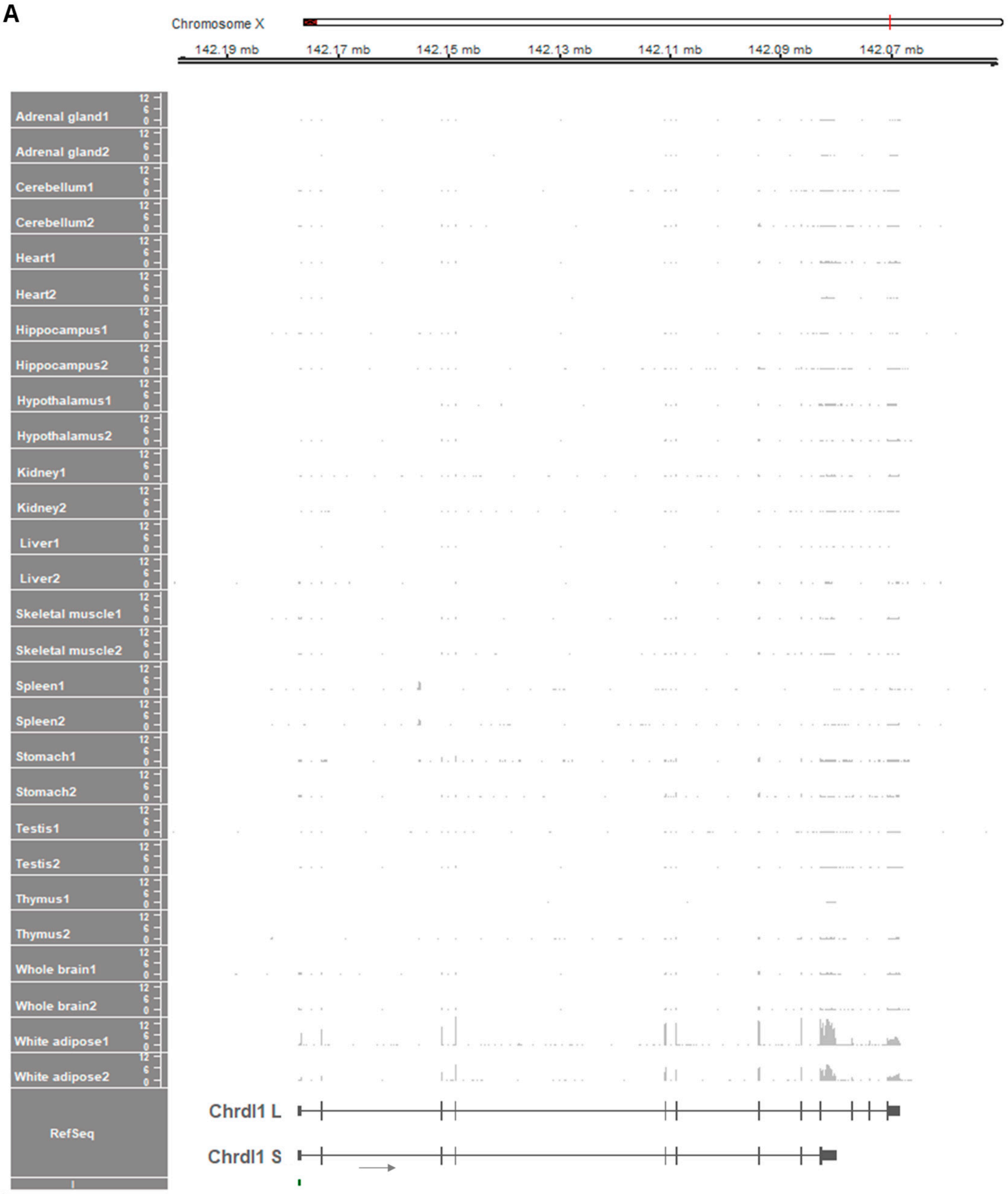

Chromosome X
142.19 mb
142.17 mb
142.15 mb
142.13 mb
142.11 mb
142.09 mb
142.07 mb
Adrenal gland1
Adrenal gland2
Cerebellum1
Cerebellum2
Heart1
Heart2
Hippocampus1
Hippocampus2
Hypothalamus1
Hypothalamus2
Kidney1
Kidney2
Liver1
Liver2
Skeletal muscle1
Skeletal muscle2
Spleen1
Spleen2
Stomach1
Stomach2
Testis1
Testis2
Thymus1
Thymus2
Whole brain1
Whole brain2
White adipose1
White adipose2
RefSeq
Chrdl1 L
Chrdl1 S

B

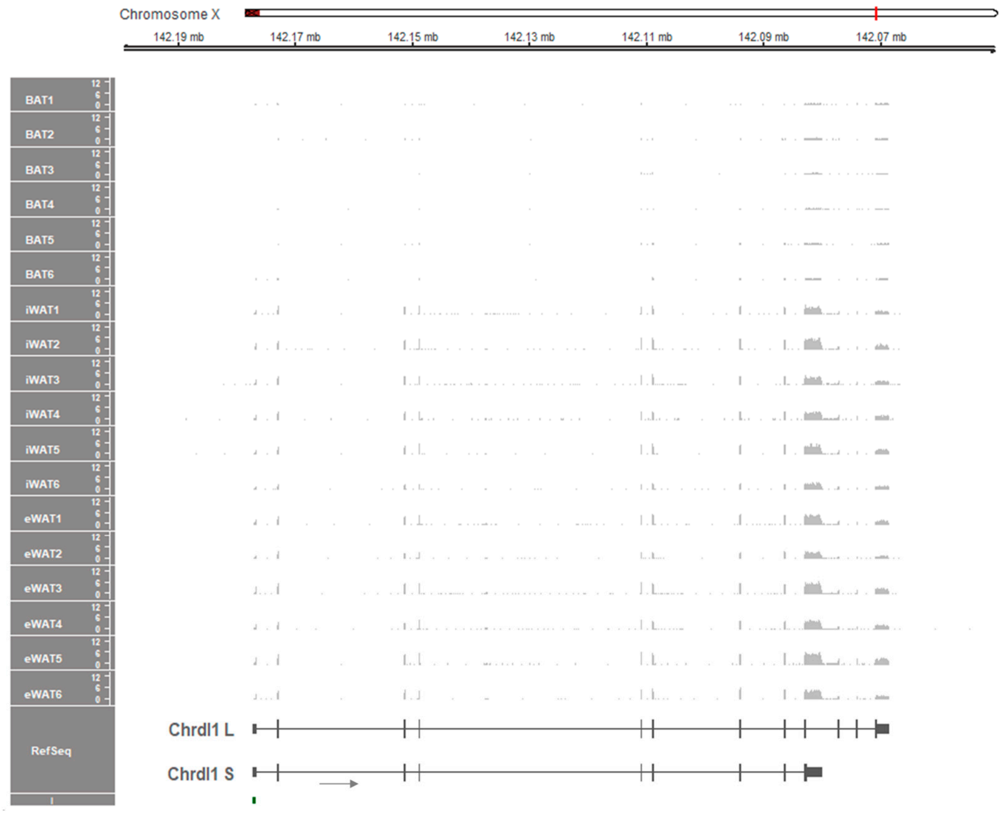

Figure S3. Expression levels of mouse *Chrdl1* mRNA. (A) Mouse *Chrdl1* mRNA expression in various tissues based on RNA-seq data (NCBI SRA study SRP020526). (B) RNA-seq read coverages of mouse *Chrdl1* in BAT, iWAT, and eWAT (GSE131861). TPM values are presented on the y-axis. These read coverages are averaged in Figure 1B. RefSeq, NCBI RefSeq gene; I, GpG island.

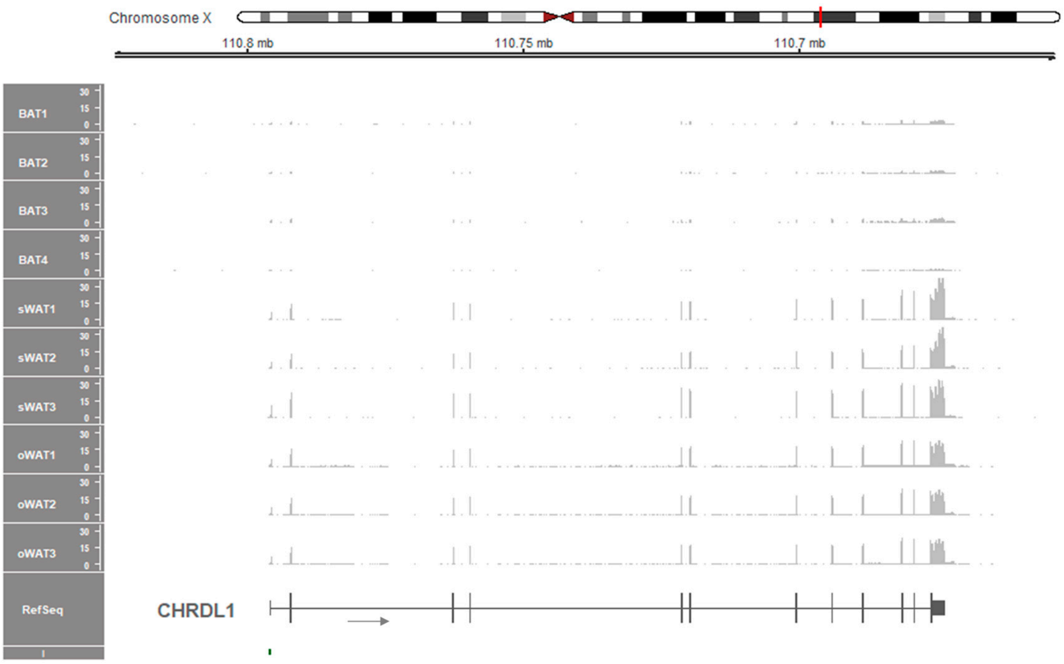

Figure S4. Expression levels of human *CHRDL1* mRNA. RNA-seq read coverages of human *CHRDL1* in BAT, oWAT, and sWAT. The y-axis represents TPM values. These read coverages are averaged in Figure 2B. RefSeq, NCBI RefSeq gene; I, GpG island.

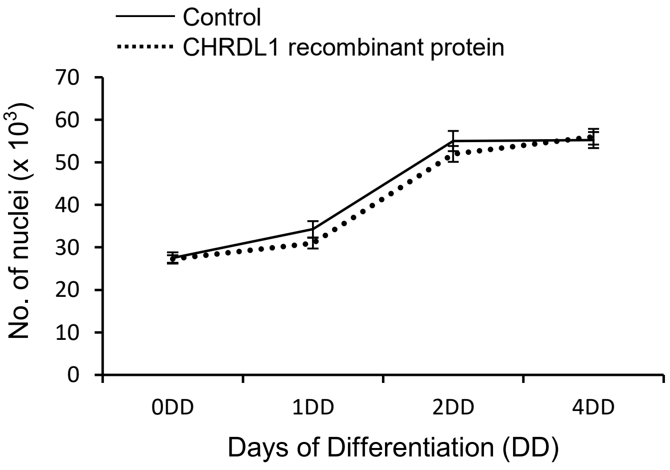

Figure S5. Measurement of cell numbers. Changes in numbers of nuclei in control and CHRDL1 recombinant protein-treated 3T3-L1 cells at 0, 1, 2 and 4 days of differentiation (DD). The Image-based Tool for Counting Nuclei (ITCN) was used for counting after DAPI staining. Values are represented as means ± SEM (n = 4). There was no significant difference between the groups at each time point.
